# ANO1 amplification and expression in HNSCC with a high propensity for future distant metastasis and its functions in HNSCC cell lines

**DOI:** 10.1038/sj.bjc.6605823

**Published:** 2010-07-27

**Authors:** C Ayoub, C Wasylyk, Y Li, E Thomas, L Marisa, A Robé, M Roux, J Abecassis, A de Reyniès, B Wasylyk

**Affiliations:** 1 Institut de Génétique et de Biologie Moléculaire et Cellulaire, UMR 7104 CNRS UDS – U 964 INSERM, 1 Rue Laurent Fries, BP 10142, 67404 Illkirch cedex, Graffenstaden, France; 2 Programme Cartes d’Identité des Tumeurs (CIT), Ligue Nationale Contre le Cancer, 14 Rue Corvisart, F-75013, Paris, France; 3 Centre Régional de Lutte Contre le Cancer Paul Strauss, Laboratoire de Biologie Tumorale, 3 Rue de la porte de l’Hôpital, F-67085 Strasbourg Cedex, France

**Keywords:** HNSCC, distant metastasis, ANO1, CaCC, cell movement

## Abstract

**Background:**

Head and neck squamous cell carcinoma (HNSCC) is associated with poor survival. To identify prognostic and diagnostic markers and therapeutic targets, we studied ANO1, a recently identified calcium-activated chloride channel (CaCC).

**Methods:**

High-resolution genomic and transcriptomic microarray analysis and functional studies using HNSCC cell line and CaCC inhibitors.

**Results:**

Amplification and overexpression of genes within the 11q13 amplicon are associated with the propensity for future distance metastasis of HPV-negative HNSCC. ANO1 was selected for functional studies based on high correlations, cell surface expression and CaCC activity. ANO1 overexpression in cells that express low endogenous levels stimulates cell movement, whereas downregulation in cells with high endogenous levels has the opposite effect. ANO1 overexpression also stimulates attachment, spreading, detachment and invasion, which could account for its effects on migration. CaCC inhibitors decrease movement, suggesting that channel activity is required for the effects of ANO1. In contrast, ANO1 overexpression does not affect cell proliferation.

**Interpretation:**

ANO1 amplification and expression could be markers for distant metastasis in HNSCC. ANO1 overexpression affects cell properties linked to metastasis. Inhibitors of CaCCs could be used to inhibit the tumourigenic properties of ANO1, whereas activators developed to increase CaCC activity could have adverse effects.

Head and neck squamous cell carcinoma (HNSCC) is common worldwide and is associated with poor survival (Parkin *et al*, 1999, 2005; Dobrossy, 2005), mainly due to relapse, metastasis and second cancer (Forastiere *et al*, 2001; Le Tourneau *et al*, 2005). Owing to the increasing incidence of HNSCC, there is a growing need for new prognostic and diagnostic markers and therapeutic targets, as well as a better understanding of the biological functions that contribute to clinical outcome. We have studied changes in gene expression in HNSCC tumours compared with histologically normal matched tissues, using differential display and microarray analysis (Lemaire *et al*, 2003; Carles *et al*, 2006). This led to the identification of a novel uncharacterised gene located on chromosomal region 11q13 that is overexpressed in HNSCC, which is now called anoctamin 1 (*ANO1*, also called *TMEM16A*, *ORAOV2*, *DOG1*, *TAOS2* and *FLJ10261*). We showed that *ANO1* is overexpressed at the RNA and protein levels in HNSCC, predicted that ANO1 is a membrane protein, and also suggested that it might be a good candidate for targeted anticancer therapy (Carles *et al*, 2006). In later studies, we compared tumours from patients with different clinical outcomes (Rickman *et al*, 2008; Jung *et al*, 2010). The propensity for subsequent distant metastasis in HNSCC was analysed using primary tumours from patients initially treated by surgery that developed (M) or did not develop (NM) metastases as the first recurrent event. Transcriptome (Affymetrix HGU133_Plus2 (Paris, France), quantitative reverse transcriptase PCR) and array comparative genomic hybridisation analysis identified transcripts that were significantly associated with M/NM status, as well as genomic areas including 11q13. However, the genomic analysis was of low resolution, precluding the identification of changes at the single gene level that could be linked to individual transcripts (Rickman *et al*, 2008). We report here a reanalysis of the same samples with genomic microarrays (Illumina 370K SNP, Paris, France) that provided individual gene resolution, which rekindled our interest in ANO1.

*ANO1* consists of 26 exons and has been predicted to code for a variety of proteins. It belongs to a protein family with eight transmembrane helices and N- and C-termini that face the cytoplasm (Katoh and Katoh, 2004; Galindo and Vacquier, 2005). ANO1 has two conserved domains of unknown function, a domain that could interfere in meiotic segregation, and multiple potential glycosylation and phosphorylation sites (Katoh and Katoh, 2003). ANO1 has recently been reported to function as a calcium-activated chloride channel (CaCC) (Caputo *et al*, 2008; Schroeder *et al*, 2008; Yang *et al*, 2008). It is highly expressed in tissues with high CaCC activity, and is required for normal development of the trachea (Rock *et al*, 2008). Chloride channels are relatively underexplored for drug discovery. They have roles in diverse physiological processes, such as fluid secretion, olfactory perception, and neuronal and smooth muscle excitability. Mutations in these channels cause human diseases, including cystic fibrosis, and have applications in treating disorders, such as hypertension and others. There are many opportunities for chloride channels in drug discovery, including, for example, increasing ANO1 activity to treat cystic fibrosis (Verkman and Galietta, 2009).

The *ANO1* gene is located on 11q13 (Katoh and Katoh, 2003), a chromosomal region that is frequently amplified in HNSCC and is associated with poor outcome (for reviews, see Gollin, 2001; Katoh and Katoh, 2003; West *et al*, 2004; Perez-Ordonez *et al*, 2006; Espinosa *et al*, 2008; Haddad and Shin, 2008). Furthermore, *ANO1* is one of a cassette of genes that have been suggested to drive 11q13 amplification by providing growth or metastatic advantage to cancer cells (Huang *et al*, 2006). In this study, we report that amplification and overexpression of *ANO1* is highly correlated with the future development of metastasis in HPV-negative HNSCC. ANO1 is involved in cell motility, invasion and adhesion of HNSCC cells, which could account for this clinical association. Inhibitors of CaCC activity inhibit ANO1-induced migration, suggesting that CaCC activity is important for cell movement. These results raise the possibility that CaCC inhibitors could be explored for tumour therapy.

## Materials and methods

### Patients and samples

Tumour samples were collected from the Biological Collection of the Centre Paul Strauss. Patients were operated for primary HNSCC between 1988 and 2003. Tumour samples were collected at the time of surgery, with the patient's informed consent. A fragment was taken near the advancing edge of the primary tumour (avoiding its necrotic centre), immediately frozen in liquid nitrogen and stored at −80°C. The rest of the tumour was fixed in 6% buffered formaldehyde and embedded in paraffin for histopathological analysis. The UICC TNM system (Staaf *et al*, 2008) was used for tumour-node-metastasis staging. Histological examination of sections adjacent to each tumour fragment showed that 60–80% were tumour cells.

A total of 83 samples were included in genome array analysis. The criteria for inclusion were tumour localisation (oral cavity, tongue, oropharynx and hypopharynx), no detectable human papillomavirus genome, no clinically evident distant metastases by conventional clinical and diagnostic radiological examinations (computed tomography), surgical resection was the first treatment, and at least 3 mm of the surgical margins were histologically tumour free. During the clinical follow-up, they either developed distant metastases to the bone, lungs or liver as the first recurrence (M, 39 cases) or did not (NM, 45 cases) (Supplementary Table 3).

### Genomic transcriptomic and membrane topology analysis

Genomic profiles were obtained using Illumina 370K SNP chips (*n*=80; Arrayexpress E-TABM-994, http://www.ebi.ac.uk/microarray-as/ae) and 4.7K CIT-CGH BAC arrays (*n*=83; Arrayexpress E-TABM-995 dataset). Transcriptomic profiles (*n*=83) of the same set of samples were previously described (Rickman *et al*, 2008) (Arrayexpress E-TABM-302 dataset). Pre-treatment steps are described in detail in the Supplementary Materials and Methods.

### Cells

HEp-2 clones were established by calcium phosphate transfection with expression vectors for ANO1 or the corresponding empty vector (pSG5-puromycin), selection for 18 days with 2 *μ*g ml^–1^ puromycin and isolation of individual clones. siRNAs were transfected with lipofectamine (Invitrogen, Cercy-Pontoise, France) for HEp-2 and lipofectamine 2000 (Invitrogen) for SCC-25.

### Western blots

Cell lysates were made up to 1 × Laemli buffer, heat denatured, fractionated by 10% SDS polyacrylamide gel electrophoresis, transferred to nitrocellulose membranes, and revealed with antibodies (ANO1: PAb2069, 1/1000 (Carles *et al*, 2006); TBP: MAb3G3, 1/2000, (IGBMC)), followed by HRP-conjugated secondary antibodies and the enhanced chemiluminescence kit (Pierce, Illkirch, France).

### Reverse transcriptase quantitative PCR

RNA was extracted with the GeneElute mammalian total RNA miniprep kit (Sigma-Aldrich, Lyon, France), verified by agarose gel electrophoresis for integrity, reverse transcribed with Superscript II (RTase SC, Life Technologies) and oligodT primer (Sigma-Aldrich), and amplified by quantitative PCR using a LightCycler and LC Fast start DNA master SYBR green I kit (Roche Diagnostics, Meylan, France). Quantitative PCRs for each experiment were carried out at least twice. The primers were designed by primer3 software (http://frodo.wi.mit.edu/primer3/). PCR specificity was verified by melting curve analysis and agarose gel electrophoresis. Standard curves were prepared using different dilutions of cDNA for each pair of primers, and expression levels were normalised with RPLP0.

### Proliferation assays

Proliferation was measured with MTT (Chemicon International, Inc., Molsheim, France). In all, 2000 cells per well were seeded in 96-well plates, and six wells were averaged for each time point. Growth in soft agar (0.35% agarose, DIFCO Laboratories, Le Pont de Claix, France) was analysed by plating 5000 cells per 35 mm Petri dish on 1% agarose. Several drops of complete medium were added to the plates once a week and, after 2–3 weeks, foci were stained with 0.005% crystal violet, photographed with a Nikon Coolpix 995 (Paris, France) and analysed using Image J (http://rsb.info.nih.gov/ij/). Each clone was plated in quadruplet per experiment.

### *In vitro* wound healing and time lapse microscopy

Cells were plated in duplicate in 24-well plates (Becton Dickinson, Le Pont de Claix, France; ref. 353047), grown to confluence, scraped with 200-*μ*l disposable plastic pipette tips and washed with phosphate-buffered saline. In some experiments, 5 or 10 *μ*M aphidicolin (Sigma) was added before wounding. Images were collected every 20 min for 48 h with an inverted microscope (Leica DMRIB (St Jorioz, France), magnification × 40, Hoffman contrast), a Cool Snap FX camera and Metamorph software (Universal Imaging, Evry, France). The distances between the wound edges were measured using Adobe Photoshop CS2. To study ANO1 inhibition, 60–80% confluent cells were transfected with siRNA (25 nM, HEp-2 clones; 50 nM, SCC-25 cells), grown to confluence (24–48 h after transfection) and then wounded. To study the effects of pharmacological inhibitors, the cells were seeded (1.2 × 10^5^) in 24-well plates, grown to confluence, wounded and washed with phosphate-buffered saline. The wounded monolayers were then incubated with the compounds or the solvent (DMSO 0.1%) and photographed after 0, 8, 24, 36 and 48 h.

### Boyden chamber migration and invasion assays

Cell migration (on collagen I or BSA) and invasion (Matrigel plug) assays were performed according to the manufacturer's instructions (collagen quantitative cell migration assay; Chemicon International, Inc). The cells were plated in duplicate per experiment.

### Cell adhesion, spreading and detachment assay

To measure adhesion, cells were plated (10^6^ cells per well in 6-well plates), incubated for 10 min at 37°C, and adherent and non-adherent (collected with two phosphate-buffered saline washes) cells were counted. The cells were plated in duplicate per experiment. Cell spreading and detachment assays were performed as described by Rodrigues *et al* (2005) and Tchou-Wong *et al* (2006), respectively.

### Statistical analysis

Statistical significance was assessed using Student's *t*-test (Microsoft Excel, Redmond, WA, USA). Univariate Cox models were calculated using the *coxph* function from the survival R package (http://www.r-project.org/). Genome × transcriptome correlations were calculated using the Pearson coefficient of correlation between the log 2 expression intensities of a probe set and the logRratios of an snp probe.

### Further details

See the Supplementary Materials and Methods.

## Results

### ANO1 amplification and overexpression are associated with distant metastasis

**Figure 1:**
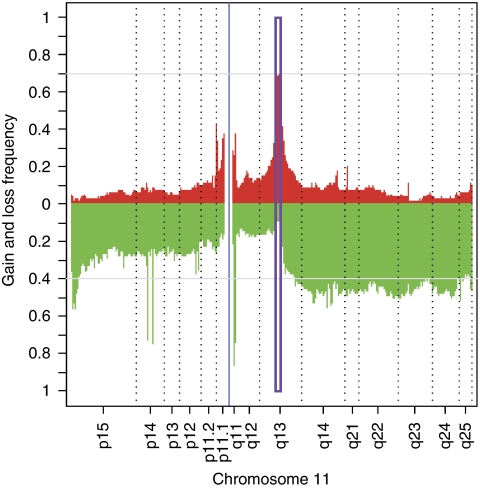
Genomic and transcriptomic microarray analysis of head and neck squamous cell carcinoma (HNSCC) patient samples. Frequency of gains and losses of chromosome 11 sequences plotted along the chromosome. Single-nucleotide polymorphism (SNP) array data (GNL: gain/normal/loss) were used to identify gains (red) and losses (green) along chromosome 11. The boxed area indicates the predominant peak of amplification corresponding to the 11q13 amplicon. The colour reproduction of the figure is available on the html full text version of the paper.

To study DNA copy-number changes in HNSCC tumours, we used high-resolution genomic microarrays (Illumina 370K SNP) to analyse 80 different samples. We detected a distinct peak of frequent genomic gain at 11q13 (Figure 1). As copy-number gains can lead to increases in gene expression, we compared these gains with RNA levels that were determined for the same patients (Affymetrix U133 plus 2.0 arrays; (Rickman *et al*, 2008)). There is a correlation between expression levels and DNA copy numbers for some of the genes in the 11q13 amplicon (see Table 1), as expected, if copy-number gains increase expression. The correlation was particularly high for some of the genes in the amplicon (*PPFIA1*, *OROAV1*, *CCND1*, *FADD*, *TPCN2*, *ANO1* and *CTTN*), suggesting that high-level expression of these genes could be functionally important.

Amplification and expression could be important for clinical outcome. Our patient group allowed us to study the association of amplification and expression with the development of future distant metastasis (M/NM status). Using Cox univariate analysis of the array data, we found that both amplification and expression of some of the genes in 11q13 are correlated with M/MN status. There is a particularly strong correlation between amplification and M/MN status for probes located within or close to *SHANK2*, *FGF4* and *ANO1* (see Table 1). For *ANO1*, the snp probe with the lowest log-rank *P*-value had a relative risk of 4.3 (see Supplementary Table 1a). There was also a strong correlation between RNA expression and M/NM status for several genes, including *FGF19*, *FGF4*, *CCND1*, *FADD*, *ORAOV1* and *ANO1* (see Table 1 and Supplementary Table 2). We reasoned that the genes with the highest correlations could be particularly important functionally. Using a series of cutoffs for selection, we found that ANO1 and ORAOV1 had the best correlations (Table 1). ANO1 was selected for further study because it is a membrane protein that is exposed at the surface of the cell, which might potentially be used for the development of antibody-based tumour therapies. Since this choice was made, the Tumorscape portal (http://www.broadinstitute.org/tumorscape, (Beroukhim *et al*, 2010; Bignell *et al*, 2010)) proposed that ANO1 and FADD are the best candidate genes in oesophageal squamous cell carcinoma for the 11q13 amplicon.

### ANO1 overexpression in stably transfected HEp-2 cells does not affect cell proliferation and growth in soft agar

**Figure 2:**
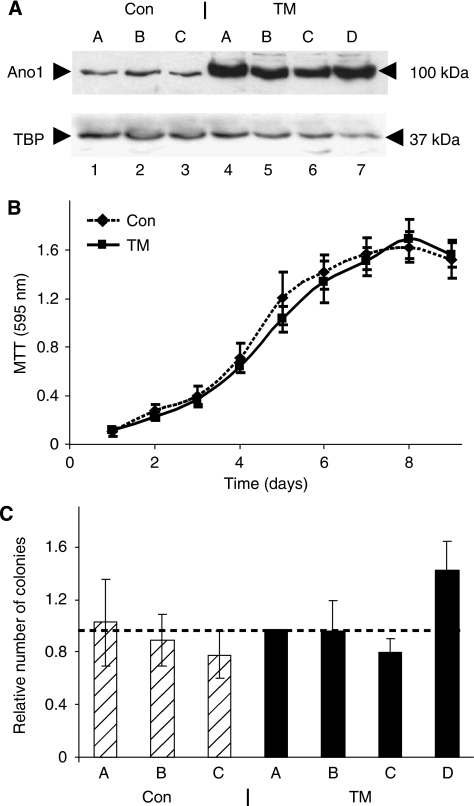
ANO1 overexpression does not affect cell proliferation and growth in soft agar. (**A**) ANO1 overexpression in stably transfected HEp-2 cells. Extracts of isolated clones (lysis buffer I) were analysed by western blotting using the anti-ANO1 antibody. TM-A, TM-B, TM-C and TM-D: ANO1-overexpressing clones; A, B and C: control clones established using the corresponding empty vector. (**B**) Cell proliferation measured by the MTT (3-(4,5-Dimethylthiazol-2-yl)-2,5-Diphenyltetrazolium Bromide) assay for the three control (dotted line) and four TM (solid line) clones. MTT conversion was measured at 595 nm. This experiment was repeated three times and similar results were obtained (four independent experiments). (**C**) Colony formation in soft agar. The number of colonies in each experiment were normalised to TM-A. The Student's *t*-test for the control clones (hatched) and the ANO1-overexpressing clones (black) gave a *P*-value of 0.06, which we consider to be not significant. This experiment was repeated once (i.e., two independent experiments) and similar results were obtained.

To investigate the effects of ANO1 overexpression on tumourigenesis, we used the human HEp-2 cell line, which was established from a laryngeal epidermoid carcinoma and expresses low levels of ANO1 compared with the other HNSCC cells lines that we tested (data not shown). We established stable HEp-2 clones that express ANO1 and control clones with the corresponding empty vector. Western blot analysis of the clones revealed a band at the expected size of 100 KD that was more intense in the four clones transfected with the ANO1 expression vector (TMEM16A, abbreviated to TM A–D; Figure 2A, lanes 4–7) compared with the three control clones (Con A–C, lanes 1–3). To examine the effects of ANO1 overexpression on cell proliferation, we performed MTT assays. ANO1 overexpression did not significantly affect cell growth in normal (Figure 2B) or in low serum conditions (data not shown). We then studied the effects of ANO1 overexpression on growth in soft agar. There was no statistically significant difference in colony formation between the groups of three control clones and four ANO1-overexpressing clones (Figure 2C).

### ANO1 overexpression stimulates migration and invasion of HEp-2 cells

**Figure 3:**
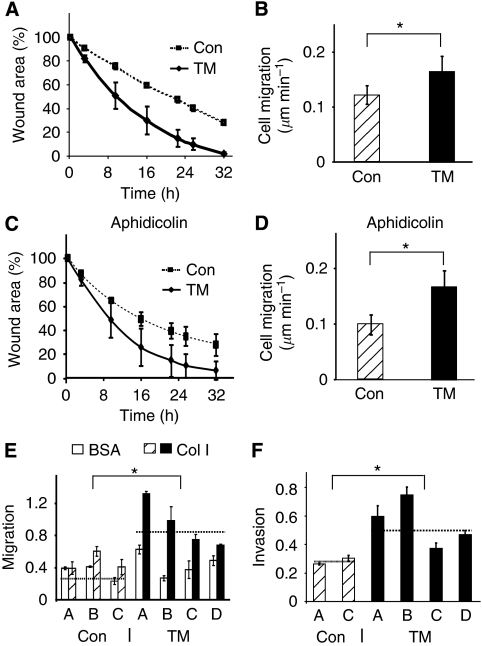
ANO1 overexpression stimulates cell migration and invasion. (**A**, **C**) *In vitro* wound healing assays in the absence (**A**) or presence of 5 *μ*M aphidicolin (**C**). The mean values were calculated for the control clones (dotted line) and for the overexpressing clones (solid line). (**B**, **D**) Rate of individual cell migration. The distance covered by individual cells was measured at different times of wound healing using Image J. The *P*-value between the controls (Con) and the overexpressing clones (TM) was ⩽2 × 10^−05^. (**E**) Boyden chamber migration assay. The cells that migrated through the porous membranes coated with BSA or collagen I (Col I) were quantified 2.5 h after seeding. The results are the averages from two independent experiments (Student's *t*-tests: BSA, *P*=0.35; Col I, *P*=2 × 10^−4^). (**F**) Boyden chamber invasion assay. Invading cells were quantified 5.5 h after seeding. The results are the averages from two independent experiments (Student's *t*-test, *P*=0.002). The dotted lines in (**E**) and (**F**) indicate the average values for each group of stable clones. ^*^Indicates statistical significance (*P*-value <0.05).

We used *in vitro* wound assays to study cell migration. Wound closure by the ANO1-overexpressing clones was faster than the controls throughout the time course of the experiment (Figure 3A). To follow individual cell migration, we measured the distance covered by separate cells at different time points (Figure 3B). ANO1-overexpressing cells migrated with an average speed of 0.16 *μ*m min^–1^ compared with 0.12 *μ*m min^–1^ for the controls; an increase of ∼25%. The difference between the two groups of clones was significant (Student's *t*-test, *P*⩽2 × 10^−05^). To eliminate the contribution of cell division to wound closure, we used 5 *μ*M aphidicolin, which decreased DNA synthesis by >95% (data not shown). Wound closure by the TM clones remained faster in the presence of aphidicolin (Figure 3C). Furthermore, individual ANO1-overexpressing cells in the presence of aphidicolin migrated with an average speed of 0.16 *μ*m min^–1^ compared with 0.1 *μ*m min^–1^ for the controls; an approximate increase of 40% (Figure 3D). These results show that ANO1 overexpression increases cell migration, which could account for the faster wound closure.

To study the effects of different substrates on migration, we used Boyden chambers coated with collagen I or BSA. ANO1-overexpressing clones migrated about two times faster than the control clones across porous membranes coated with collagen I (Figure 3E). Interestingly, there was no significant difference when the membranes were coated with BSA (Figure 3E), suggesting that interactions with collagen I are important for the effects of ANO1 on migration. We also investigated whether ANO1 overexpression could affect migration through Matrigel plugs in Boyden chambers. We found that invasion by the TM clones was about 50% greater than the control clones (Figure 3F), showing that ANO1 overexpression stimulates cell migration through Matrigel.

### ANO1 overexpression affects cell adhesion, spreading and detachment

**Figure 4:**
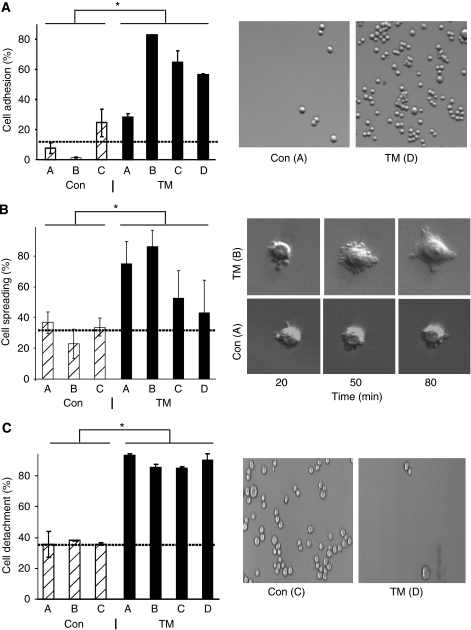
ANO1 overexpression affects cell adhesion, spreading and detachment. (**A**) ANO1 increases cell adhesion. Left panel: the values are the means of two independent experiments. Student's *t*-test, Con *vs* TM, *P*=2 × 10^−04^. Right panel: representative photos of cell attachment of control clone A and overexpressing clone TM-D. (**B**) ANO1 increases cell spreading. Each clone was analysed by time-lapse microscopy at four different positions of the camera. The data represent the proportion of cells that had spread 2 h after plating and correspond to the mean value of three independent experiments. Student's *t*-test, Con *vs* TM, *P*=7 × 10^−07^. Right panel: representative photos of clone A and clone TM-B cells at different times after plating. (**C**) TM increases cell detachment. The results are the averages from two independent experiments. Student's *t*-test, Con *vs* TM, *P*=5 × 10^−11^. Right panel: representative photos of cell detachment for clone C and clone TM-D. The dotted lines indicate the average values for the control clones. ^*^Indicates statistical significance (*P*-value <0.05).

We investigated cellular properties that could affect cell migration, including attachment, spreading and detachment. To measure attachment, cells were plated at the same density and incubated for 10 min. In conditions in which 30–80% of the cells of ANO1-overexpressing clones attached to the plate surface, <25% of the control clone cells attached (Figure 4A, representative photomicrographs are shown for clones with median properties within their group). These results show that ANO1 overexpression increases cell attachment. We then investigated cell spreading. Cells that overexpress ANO1 began to spread and form lamellipodia significantly faster than the controls (Figure 4B, representative cells are shown). The increase was on an average about 50% greater for the ANO1-overexpressing cells, at 2 h after plating. To analyse detachment, we treated the cells with trypsin. An average of 88% of the cells detached in the case of the ANO1-overexpressing clones, compared with 36% for the controls (an increase of about 40%, Figure 4C). Taken together, these results demonstrate that ANO1 affects cell adhesion, spreading and detachment in a manner that is favourable for migration.

### ANO1 inhibition decreases cell migration in an ANO1-overexpressing clone

**Figure 5:**
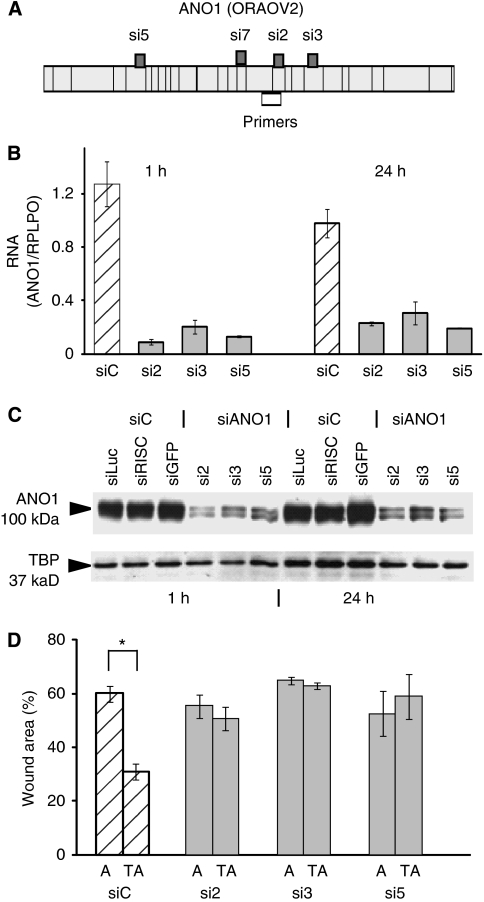
Anoctamin 1 (*ANO1*) silencing decreases cell migration in the TM-A clone. (**A**) Schematic representation of the exon structure of ANO1 (ORAOV2), and the localisations of siRNAs 2, 3 and 5, and of the primers used for reverse transcriptase quantitative PCR (RT–qPCR). The primers target all the known *ANO1* transcripts. (**B**) RT–qPCR analysis of *ANO1* mRNA expression in clone TM-A transfected with control siRNAs (siC), and siRNAs 2, 3 and 5. At 24 h after transfection, the cells were scrape-wounded, and after a further 1 h and 24 h, RNA was extracted and analysed by RT–qPCR. The siRNA control (SiC) is the average for siLuc, siRISC and siGFP. The data are the mean of two independent experiments. (**C**) Western blot analysis of ANO1 protein expression in clone TM-A following transfection with the indicated siRNAs, 1 h and 24 after scrape wounding. Cell extracts were prepared with lysis buffer II. (**D**) Wound closure following siRNA transfection of control clone A and TM-A (TA). Wound areas were measured 12 h after scraping. The graph represents the average of two independent experiments. ^*^Indicates statistical significance (*P*-value <0.05).

We studied whether the effects of stable ANO1 overexpression in the isolated clones could be transiently overcome with *ANO1* siRNAs (si2, si3 and si5, Figure 5A). The cells were transfected with control siRNAs (siC), si2, si3 and si5, allowed to reach confluence (24–48 h later), wounded with multiple scrapes in a crisscross manner, and after 1 and 24 h harvested and used to analyse ANO1 expression by reverse transcriptase quantitative PCR and western blotting. The ANO1 siRNAs specifically decreased *ANO1* RNA (Figures 5B) and protein (Figure 5C) expression, and the decrease was similar at the beginning and end of the wound-healing experiment (1 and 24 h after wounding). Downregulation of ANO1 decreased wound closure to the level observed with the control clone (Figure 5D). Wound closure was not significantly altered in the control clone, as would be expected from the low level of endogenous ANO1 expression in these cells (Figure 2A and data not shown). These results indicate that the siRNAs do not have off-target effects, and that exogenous ANO1 is involved in wound closure.

### Silencing endogenous ANO1 decreases the migration of SCC-25 cells

**Figure 6:**
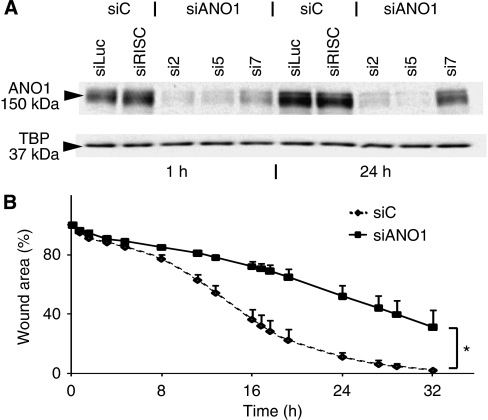
Inhibition of endogenous expression of ANO1 decreases cell migration. (**A**) Transfection of siRNAs 2, 5 and 7 inhibits ANO1 protein expression in SCC-25 cell line. Cells transfected with siRNAs for 48 h were scrape-wounded. Proteins were extracted 1 and 24 h after cell injury and were analysed by western blotting. (**B**) ANO1 inhibition decreases migration of SCC-25 cells – shown by *in vitro* wound healing on SCC-25 transfected with siRNAs (*N*=2). ^*^Indicates statistical significance (*P*-value <0.05).

To study the effects of silencing endogenous ANO1 on cell migration, we used SCC-25 squamous cell carcinoma cells that express a higher level of ANO1 than HEp-2. *ANO1* knockdown during the wound-healing assay was analysed by western blot analysis (Figure 6A). siRNAs 2, 5 and 7 (see Figure 5A for positions) specifically decreased the levels of ANO1. The migration of the specific band (apparent molecular weight of 150 KDa) is slower than the form expressed in HEp-2 (Figure 2A), suggesting dissimilar folding of this hydrophobic membrane protein during SDS polyacrylamide gel electrophoresis. Knockdown of endogenous *ANO1* in SCC-25 decreased wound closure throughout the time course of the experiment (Figure 6B). These results reinforce the overexpression data, and show that ANO1 is involved in wound closure and movement.

### CaCC blockers decrease cell migration in ANO1-overexpressing clones

**Figure 7:**
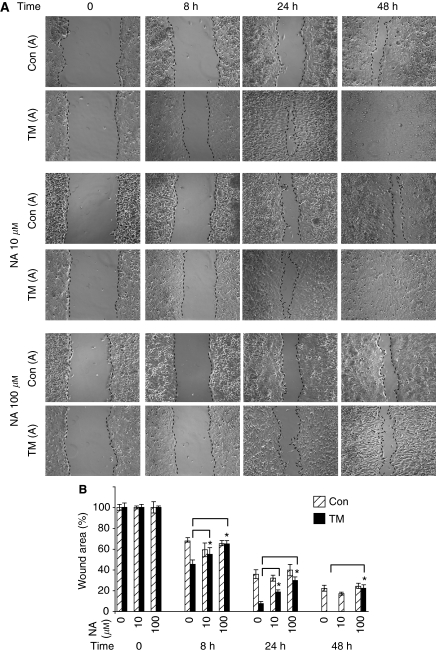
Effect of niflumic acid (NA) on wound closure induced by ANO1. Confluent monolayers of TM-A-, TM-B- and TM-C-overexpressing clones and control clones A, B and C (Con) were wounded with a sterile pipette tip, incubated with NA or solvent alone, and photomicrographs were taken at different times with an inverse microscope (Leica) and the Cool Snap System (Photometrics, Göttingen, Germany). (**A**) Representative photomicrographs for the clones TM-A and Con-A after 0, 8, 24 and 48 h. (**B**) Graphical representation of wound area as a function of time and NA concentration. Average values were calculated for three ANO1-overexpressing (TM-A, -B and -C) and three control clones. The difference between the treated (NA in 0.1% dimethylsulphoxide (DMSO)) and untreated (0.1% DMSO) cells was calculated for each group of clones (TM and Con). The results are the average of three independent experiments and two independent woundings for each clone. ^*^Student's *t*-test, *P*<0.02.

**Figure 8:**
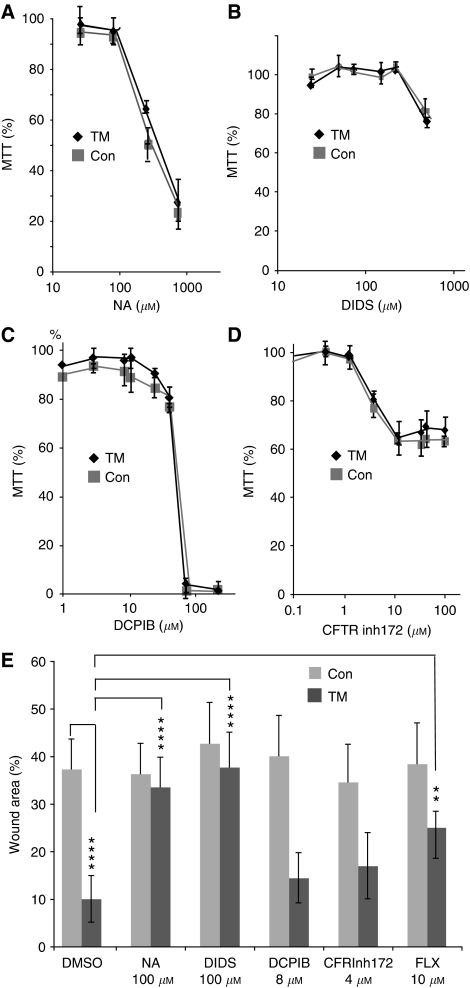
IC10 (inhibitory concentration of 10%) determination and effects on wound closure of pharmacological inhibitors. (**A–D**) The Con A–C and TM-A–C clones were seeded at 50 000 cells per well in 96-well plates, incubated with various concentrations of the indicated compounds for 48 h, and then analysed by the MTT assay. The values are the averages for six wells for each of the three clones of a particular type (Con or TM), carried out twice in two experiments. The error bars indicate the s.d. The IC10 values were determined with Matlab software (The Mathworks, Meudon, France). (**E**) Confluent monolayers of TM clones A, B and C, and Con clones A, B and C were wounded, incubated with the indicated compounds or solvent alone (dimethylsulphoxide (DMSO)), and photomicrographs were taken at different times with an inverse microscope (Leica) and the Snap Cool system. The values shown are for 36 h, four areas for each wound, two wells per clone, averaged over the three Con or TM clones. The wound area is expressed relative to the zero time point. One representative experiment of four is shown. Consistent results were obtained at different time points (24, 36, 48 and 72 h; data not shown). The error bars represent the s.d. The number of stars indicate the *P*-value exponents from Student's *t*-tests; ^**^10^−2^, ^***^10^−3^ and ^****^10^−4^.

To investigate whether ANO1 channel activity is required for cell migration, we used the inhibitor niflumic acid (NA) (Caputo *et al*, 2008; Schroeder *et al*, 2008; Yang *et al*, 2008). Concentrations of 10 and 100 *μ*M NA delayed wound filling by the ANO1-overexpressing cells compared with untreated cells, whereas they had little effect on the control clones (Figure 7A; the photographs show TM-A and the control clone A; Figure 7B; shows the average values for the groups of TM and control clones). The lack of an effect on the control clones is expected, as they expressed low levels of ANO1. Furthermore, it indicates that the effect of NA is specific for ANO1. To investigate the pharmacological profile of inhibition, we tested DIDS, which inhibits the CaCC activity of ANO1 ((Schroeder *et al*, 2008; Yang *et al*, 2008), and as negative controls DCPIB (a fairly specific inhibitor of volume-sensitive chloride channels) and CFTRinh172 (a selective inhibitor of CFTR). We first determined their IC10 values using MTT assays under the conditions used for wound closure (freshly confluent cells, 48 h; Figures 8A–D). The IC10 values for each compound were similar for the control and ANO1-overexpressing clones (NA 100±20 *μ*M, DIDS 300±50 *μ*M, DCPIB 10±3 *μ*M; CFTRinh172 5±2 *μ*M), showing that there was little variation in sensitivity between the clones. Using concentrations of the compounds close to the IC10 (NA, DCPIB and CFTRinh172) or lower (DIDS, 100 *μ*M), we found that DIDS specifically inhibited wound closure of the ANO1-overexpressing clones, similar to NA, whereas DCPIB and CFTRinh172 had no significant effects (Figure 8E). In addition, the ANO1 CaCC inhibitor fluoxetine (10 *μ*M; IC10 20 *μ*M, data not shown) (Yang *et al*, 2008) also specifically inhibited the TM clones. These results suggest that CaCC activity is required for the effects of ANO1 on cell motility.

## Discussion

In this study, we show that amplification of the 11q13 locus, and *ANO1* copy number and expression are significantly augmented in patients with an increased propensity to develop metastases. ANO1 is involved in biological functions that are important for metastasis, including migration, invasion and adhesion, but apparently not for proliferation and growth in soft agar. CaCC activity in cells that overexpress ANO1 is important for cell motility.

From the analysis of 80 patient samples with SNP arrays, we found that amplification of the 11q13 locus is associated with the propensity for the development of metastasis in HNSCC. We also found this association (Cox log-rank *P*=0.05, Supplementary Table 1b) using the same patient samples in our previous BAC array-based study (Rickman *et al*, 2008), but the lower resolution (4.7K compared with 370K probes) prevented precise identification of candidate genes. Other studies have indicated that 11q13 amplification is associated with poor prognosis in HNSCC (Akervall *et al*, 1995, 1997; Klussmann *et al*, 2009) and is also associated with patient outcome in other cancers (Berenson *et al*, 1990; Kitagawa *et al*, 1991; Borg *et al*, 1991). Further studies with prospective samples will be required to evaluate whether amplification of 11q13 is a useful marker for metastatic outcome.

The high resolution of the arrays allowed us to identify SNPs within the amplicon that are associated with metastatic outcome. The highest correlations, using either log-rank test *P*-values (*P*<0.05) or relative risk, were for probes close to SHANK2, FGF4 and ANO1, and close to SHANK2, ANO1 and CTTN, respectively (Supplementary Table 1a). However, other probes throughout the amplicon were also associated with metastatic outcome, suggesting that there is no particular subregion that could be implicated in this process. These results are compatible with several other studies that used lower resolution approaches and that suggested that the whole amplicon should be considered as the functional element (Gibcus *et al*, 2007; Huang *et al*, 2006). We localised the amplicon to 68.5–70.3 Mb (HG18), in agreement with others (Huang *et al*, 2006; Gibcus *et al*, 2007; Reshmi *et al*, 2007). We also observed a high frequency of loss of 11q distal to the 11q13 amplicon, which has been proposed to drive tumourigenesis and amplification through effects on DNA repair and expression of miR-125b and miR-100 (Parikh *et al*, 2007; Henson *et al*, 2009). Increased DNA copy number in the 11q13 amplicon is associated with overexpression of several genes. We found a particularly high correlation between copy number and expression for six genes: *PPFIA1*, *ORAOV1*, *CCND1*, *FADD*, *TPCN2* and *ANO1* (in their order of correlation). A similar correlation has been reported for cell lines from oral squamous cell carcinoma (TPCN2, CCND1, ORAOV1, ANO1, FADD, PPFIA1 and EMS1 (Huang *et al*, 2006); FADD, PPFIA1 and EMS1 (Jarvinen *et al*, 2008)). The expression of the six genes identified in our study was also highly correlated with metastatic outcome: *PPFIA1*, *ORAOV1*, *TPCN2*, *CCND1*, *ANO1* and *FADD*, in the order of log-rank test *P-*values. Interestingly, 11q13 amplification has been related with nodal metastases and higher tumour recurrence rates in hypopharyngeal HNSCC (Rodrigo *et al*, 2002). EMS1 expression has been shown to predict poor survival in laryngeal carcinoma (Gibcus *et al*, 2008; Rodrigo *et al*, 2009). Some of these genes have functions that are significant for tumourigenesis, suggesting that they may drive the amplification (Martin *et al*, 2008). *CCND1*, the most studied gene, is involved in cell-cycle progression (Jares *et al*, 1994; Michalides *et al*, 1995, 1997). *PPFIA1* may regulate disassembly of focal adhesions and cell–matrix interactions (Serra-Pages *et al*, 1998). *ORAOV1* is required for cell growth and tumour angiogenesis (Jiang *et al*, 2008). *FADD*, in its phosphorylated nuclear form, is associated with increased cell proliferation (Werner *et al*, 2006). The possibility exists that several genes in the 11q13 could contribute to patient outcome (Huang *et al*, 2006), and that the other less characterised genes may also contribute. We selected ANO1 for further study, because of the correlation with 11q13 amplification and metastatic outcome, and its localisation on the cell surface. Furthermore, *ANO1* is not amplified and overexpressed in tumours that express HPV E6 and E7. Interestingly, these patients have a better prognosis (Ragin *et al*, 2006; Klussmann *et al*, 2009; Jung *et al*, 2010).

We have shown that ANO1 overexpression does not significantly affect cell proliferation during the exponential growth phase, nor anchorage-independent growth in soft agar, a common assay for tumourigenicity. Interestingly, ANO1 overexpression does stimulate migration, which is important for metastasis. We have shown that ANO1 overexpression accelerates *in vitro* wound healing on cell culture plates and movement through membranes in Boyden Chambers. Faster wound closure could have resulted from effects on cell division, spreading and movement. Increased cell proliferation did not contribute significantly, as aphidicolin (an inhibitor of DNA replication) did not have a significant effect. Furthermore, there was no effect of ANO1 overexpression on growth. Direct measurements of individual cells showed that they moved more quickly. In the Boyden chamber assays, the cells overexpressing ANO1 crossed through the pores of the membranes more quickly when they were coated with collagen I, but not when they were coated with BSA. This result suggests that the effects of ANO1 depend on attachment to the extracellular matrix. This could somehow involve integrins, the largest family of receptors that mediate cell adhesion to collagens, fibronectin and laminins (Heino and Kapyla, 2009). We have shown that ANO1 overexpression stimulates cell attachment, spreading and detachment, which are important for cell movement (Ruoslahti and Obrink, 1996; Webb *et al*, 2002). Increased movement could also account for the increased ability of overexpressing cells to migrate through Matrigel, although we cannot exclude that other mechanisms could also be involved.

We also investigated a cell line (SSC-25) that expresses a relatively high level of ANO1, which reflects the situation in the majority of HPV-negative HNSCC. Downregulation of endogenous ANO1 in SSC-25 inhibited cell movement, showing that the endogenous protein is involved in this process. This result is significant as ANO1 could be subjected to modifications that are not reproduced in the overexpressing cell clones. Furthermore, *ANO1* is a complex and poorly characterised gene with different splice variants (Ferrera *et al*, 2009). We observed, using SDS polyacrylamide gel electrophoresis, that ANO1 from SSC-25 migrated differently from the exogenously introduced protein. This may not be significant, as we found that the migration of ANO1 is quite variable depending on the conditions of extraction and denaturation. Furthermore, hydrophobic proteins have been shown to fold variably in SDS (Rath *et al*, 2009). However, we cannot exclude that the endogenous protein is somehow different. We found that three different siRNAs that target known transcripts had similar effects and gave results that were consistent with the overexpression data. This suggests that there are no major functional differences relating to cell movement between the exogenous and endogenous proteins. Some of the reported ANO1 cDNAs could code for proteins with different numbers of transmembrane domains. However, there are no reports that proteins with less than eight transmembrane segments are functional and are physiologically relevant. Recently, three splice variants have been described that affect the chloride channel properties of ANO1 (Ferrera *et al*, 2009). The significance of these new finding are interesting areas for future studies.

Our results show that amplification and overexpression of ANO1 and other genes on the 11q13 are associated with an increased frequency of future distant metastasis of HPV-negative HNSCC. ANO1 may contribute to this susceptibility in a quite specific manner, through effects on cell migration rather than growth. Additional metastatic properties could be provided by collaborating genes that are co-amplified and overexpressed, such as CCND1 for growth (Perez-Ordonez *et al*, 2006). Functional collaboration between different genes may contribute to their high frequency of co-amplification in the 11q13 locus. However, it should be strongly stressed that the data do not allow us to conclude that ANO1 (or any of the other genes) is responsible for the metastatic behaviour of patients with 11q13 amplification.

We have shown that several compounds that have been shown to inhibit the CaCC activity of ANO1 (NA, DIDS and fluoxetine (Schroeder *et al*, 2008; Yang *et al*, 2008)) specifically reduce migration of the ANO1-overexpressing cells, but not the parental clones that do not express significant levels of ANO1. Several control compounds, DCPIB (an inhibitor of volume-sensitive chloride channels) and CFTRinh-172 (a selective inhibitor of CFTR), do not have a significant effect. This suggests that the effects are due to the blockage of CaCC activity, rather than some other activity that this protein may have. However, it should be stressed that NA and the other inhibitors are not ANO1 specific (Schultz *et al*, 1999; Verkman and Galietta, 2009). Similar to our results, Mao *et al* (2005) have shown that blocking Cl^−^ ion channels inhibits migration of nasopharyngeal carcinoma cells. However, our study does not show that there is a direct correlation between the CaCC activity of ANO1 and migration, and further studies of chloride levels changes, using patch-clamp or other techniques (Bregestovski *et al*, 2009; Verkman and Galietta, 2009) will be required. The mechanisms of these processes and the contribution of chloride and calcium are interesting subjects for further study (Kunzelmann *et al*, 2009).

Considerable evidence exists for the contribution of ion channels to tumour development, growth and malignancy (Kunzelmann, 2005). Chloride channels are relatively unexplored as drug targets for many pathologies, and both inhibitors and activators are being developed (Verkman and Galietta, 2009). Our observations indicate that activators of ANO1, which may be useful in other pathological settings (e.g., compensation for the inactive CFTR chloride channel in cystic fibrosis), could potentially have undesirable effects on tumours through ANO1. Our study highlights that understanding the roles of ANO1 in cancer metastasis will be important in the rational design of drugs for this important class of targets.

## Figures and Tables

**Table 1:**
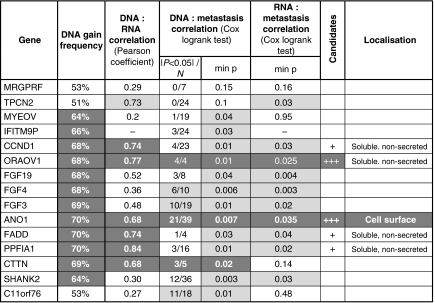
Correlation between DNA levels, RNA levels and metastasis of genes in the 11q13 amplicon
