# Supplementary material for: ANO1 amplification and expression in HNSCC with a high propensity for future distant metastasis and its functions in HNSCC cell lines
Source: Br J Cancer. 2010 Jul 27;103(5):715–26. doi: 10.1038/sj.bjc.6605823 (PMC2938263; doi:10.1038/sj.bjc.6605823)
Supplement: Supplementary Materials and Methods [file 6605823x1.doc]

**Supplementary Materials and Methods**

*Genomic and transcriptome analysis*

Except when indicated, all transcriptome and genome analysis was carried out using either an assortment of R system software ([http://www.R-project.org](http://www.R-project.org/), V2.10.0) packages including those of Bioconductor (v2.5) by Gentleman et al. (Gentlem*an et* al, 2004) or original R code.

**1**- Expression microarrays (Arrayexpress E-TABM-302 dataset)

We used the *affyQCReport* Bioconductor R package to generate a QC report for all chips. Chips that do not pass this filter were not included in this study. Raw feature data from the chips were normalized in batch using robust multi-array average (RMA) method by Irizarry et al. (Irizar*ry et* al, 2003), implemented in R package *affy*. Probe sets corresponding to control genes or having a “_x_” annotation were masked yielding a total of 50,406 probe sets available for further analyses.

2- CGHarrays (Arrayexpress E-TABM-995 dataset)

2.1- QC, filtering and normalization

Spots were filtered using the following criteria: (i) signal-to-noise < 2.0 for the reference channel, (ii) control and un-annotated spots (iii) manual flag (iv) raw intensity < 1 (v) raw intensity at saturation (=65000). The remaining raw log2ratio values were normalized using the lowess within-print tip group method from Yang et al. (Ya*ng et* al, 2002) (spots corresponding to X and Y chromosomes were masked to calculate the lowess fit). For clones (BACs/PACs) in which more than 1 feature value remained after filtering and that yielded an inter-feature standard deviation of less than 0.25, an average normalized log2-ratio value was calculated. For each chip the percentage of filtered spots and clones was calculated.

2.2- Smoothing and breakpoints detection

The normalized log2-ratio values were smoothed using the tiling Array Bioconductor package v1.24.0 (which implements the method proposed by Franck Picard et al. (Pica*rd et* al, 2005)), yielding smoothed log2-ratios values in homogeneous segments along the chromosome.

2.3- Copy number assignment

For each sample, the level (L_N_) corresponding to a normal (i.e. diploid) copy number is determined as the first mode of the distribution of the smoothed log2-ratio values across all -except sexual- chromosomes. The standard deviation (SD) of the difference between normalized and smoothed log2-ratio values is calculated. Then for all clones in a segment, the ‘GNL’ copy number status (G:gain | N:normal | L:loss) is determined as follows, based on the segment smoothed log-ratio value (X): if X > L_N_ + k x SD then status=gain (G), if X < L_N_ – k x SD then status=loss (L), else status=normal (using k=1). Outliers are classed manually and correspond to individual clones that yielded normalized log2-ratio values (Y) such that: Y > L_N_ + 3 × SD (status=gain) or Y < L_N_ - 3 × SD (status=loss).

2.4- Recurrent minimal genomic alterations

Computation of recurrent minimal genomic alterations was done in a similar way to the method described by Rouveirol et al (Rouveir*ol et* al, 2006) using original R code.

3- SNP arrays (Arrayexpress E-TABM-994 dataset)

SNP array genotyping was carried out using the Illumina “HumanCNV370-Quad” array (Illumina, Inc., San Diego, CA) on the Integragen Illumina microarray platform (Evry, France) according to the Illumina procedures. Scans were performed on the Illumina BeadArray Reader (Illumina) and data were extracted and normalized with Illumina Beadstudio software V3 by using standard settings.

3.1- Normalization

Data normalization was improved using the normalization procedure tQN proposed by Staaf et al. (Sta*af et* al, 2008b) to make allelic frequencies symmetrical.

3.2- Filtering, smoothing and breakpoints detection

To obtain one banded BAF profile, mirrored BAF was processed and non informative homozygous SNPs were removed as described in Staaf method {Staaf, 2008 #37}. The Haarseg segmentation method {Ben-Yaacov, 2008 #38} was then applied to log ratios (LRR) and allelic frequencies (mirrored BAF) data.

3.3- Copy number assignment

Segments derived from combined LRR and mirrored BAF segmentation were attributed a GNL’ copy number status (G:gain | N:normal | L:loss) as follows : for each sample, the level (L_N_) corresponding to a normal (i.e. diploid) copy number is determined as the first mode of the distribution of the smoothed LRR values across segments showing allelic balance (according to the related mirrored BAF distribution). The standard deviation (SD) of the difference between LRR and smoothed LRR values is calculated. Then for all SNP probes in a segment, the ‘GNL’ copy number status (G:gain | N:normal | L:loss) is determined as follows, based on the smoothed LRR (X): if X > L_N_ + k × SD then status=gain (G), if X < L_N_ – k × SD then status=loss (L), else status=normal (using k=0.5).

4- Genome / transcriptome correlation

We mapped SNP probes and Affymetrix probe sets based on their genomic position. Then for a pair (SNP probe, Affymetrix probe set), subgroups of samples analyzed both for transcriptome and genome were used to calculate a Pearson coefficient of correlation between the normalized log2 RMA intensity values (corresponding to the probe set) and the normalized logRratio values (corresponding to the SNP probe).

*Analysis of amino-acid sequence*

Membrane topology of ORAOV2 protein was determined by using the PSORT II program (<http://psort.nibb.ac.jp/form2.html>).

*Cell culture*

HEp-2 cells were grown in modified Eagle’s medium (MEM) containing 10% fetal calf serum (FCS) and supplemented with 1 mM sodium pyruvate, 0.1 mM AANE, 2 mM glutamine and 40 µg/ml gentamicin. SCC-25 cells were grown in DMEM/Ham’s F-12 = 1:1 media containing 10% FCS and supplemented with 40 µg/ml gentamicin and 0.4 µg/ml hydrocortisone. Cells were routinely maintained at 37°C in a humidified atmosphere of 5% CO_2_.

*Stable clones*

The MGC-33580/BC033036 in pBluescriptR (LGC Promochem) plasmid was used to clone the corresponding 8TM/ORAOV2 fragment in the pSG5 puromycin resistance vector by Expand High Fidelity PCR at Bam H1 site. The primers used were:

5’-CGGGATCCCCGATGGACTACCACGAGGA-3’ (forward)

5’-CGGGATCCTAGCTACAGGACGCCCCCG-3’ (reverse).

HEp-2 cells were transfected with calcium phosphate and expression vectors for 8TM/ORAOV2 or corresponding empty vector (pSG5 puromycin resistance plasmid). Cells were then selected for 18 days in 2 µg/ml puromycin containing medium. Individual resistant clones were picked for expansion (1 µg/ml puromycin medium) and characterisation.

*Dow-regulation by small-interfering RNA (siRNA)*

siRNA oligonucleotides targeting four different regions of *ANO1* mRNA were purchased from Dharmacon Reasearch. The target sequences were: #2: 5’-TTACGTGGCGTTCTTCAAA-3’; #3: 5’-GGTACGAGGTGGATTACAA-3’; #5: 5’-GCACGATTGTCTATGAGAT-3’; #7 5’-GAATCATTGTCTTCCTGTATT-3’.

siRNA transfection was performed using Lipofectamine (Invitrogen) for the HEp-2 cell-line and Lipofectamine 2000 (Invitrogen) for the SCC-25 cell-line, according to the manufacturer’s instructions.

*Antibodies*

The polyclonal antibody 2069 was raised by injecting rabbits with the EKERQKDEPPCNHHNTC peptide located in the C-terminal region of ANO1 (Carl*es et* al, 2006). The following antibodies were used for Western-blots at the indicated dilutions: 2069 purified antibody (1/1000); mouse anti-mouse TATA binding protein (IGBMC; TBP Ab: 3G3) (1/2000).

*Western blots*

Cells were harvested in lysis buffer I (32 mM Tris HCl [pH 6.8], 20% glycerol, 1% SDS, 5 mM dithiothreitol (DTT), 1 mM phenylmethylsulfonylfluoride (PMSF) and 1× complete protease inhibitor cocktail PIC [Roche Diagnostics]) or lysis buffer II (50 mM Tris HCl, pH 7.4, 150 mM Nacl, 1 mM EDTA, 1% TRITON^R^ X-100 supplemented with DTT, PIC and PMSF). 10× Laemli buffer was diluted to 1× in the buffer I protein lysates and samples were heated for 7 min at 52°C. Protein lysates in buffer II were centrifuged at 4°C and 10 000 rpm. 10× Laemmli buffer was diluted to 1× in each of the supernatants. Protein extracts were then fractionated by 10% sodium dodecyl sulphate-polyacrylamide gel electrophoresis, transferred to nitrocellulose membranes, and revealed with antibodies and the enhanced chemiluminescence kit (Pierce).

*Two-step Reverse Transcriptase - Quantitative Polymerase Chain Reaction (RT-qPCR)*

RNA from cultured cells was extracted using the GeneElute Mammalian Total RNA Miniprep Kit (Sigma-Aldrich). The integrity of the RNA was verified by agarose gel electrophoresis. 1 µg of total RNA was reverse transcribed to cDNA using Superscript II (RTase SC, Life Technologies) and an oligodT primer (Sigma). The relative mRNA expression levels of *ANO1* and *RPLPO* were quantified by qPCR using a LightCycler (Roche Diagnostics) with LC Fast start DNA master SYBR green I kit (Roche Diagnostics). qPCRs for each experiment were repeated minimum twice. The primers, designed by primer3 software (<http://frodo.wi.mit.edu/cgi-bin/primer3/primer3_www.cgi>), were: *ANO1*:

5’-CTCCTGGACGAGGTGTATGG-3’ (forward),

5’-GAACGCCACGTAAAAGATGG-3’ (reverse)

RPLP0 (Ribosomal Phosphoprotein Large P0):

5’-GAAGGCTGTGGTGCTGATGG-3’ (forward)

5’-CCGGATATGAGGCAGCAGTT-3’ (reverse)

The specificity of the ANO1 primers was verified by Blast analysis. Forty rounds of PCR were carried out for 10 s at 95°C, 5 s at 64 and 10 s at 72°C. The specificity of the PCR products was verified by melting curve analysis and agarose gel electrophoresis. For each pair of primers, standard curves were prepared using different dilutions of cDNA. The expression level of *ANO1* cDNA was internally normalized using RPLP0.

*MTT assay and IC10*

For growth curves, cells were seeded at a confluence of 2000 cells/well in 96-well plates, and proliferation was measured with MTT (Chemicon) according to the manufacturer’s instructions. In brief, MTT was added to the medium and incubated for 4 h. Then, isopropanol containing 0.04N HCl was added to the cells for 1 h. The absorbance was measured in an ELISA plate reader at 595 nm. The cells from each clone were plated in 6 wells per experiment. To determine IC10 values for the compounds, cells were seeded at 50 000 cells per well of a 96 well plate, 16 h later the compounds were added, incubated for 48 h, and then processed for the MTT assay.

*Cell growth analysis in soft agar*

Cells (5,000 per 35 mm Petri dish) in MEM 2× and 20% FCS were mixed with an equal volume of 0.7% agarose (DIFCO Laboratories) and poured onto a bed of 1% agarose (in HEp-2 medium). Each week, several drops of complete medium were added to the surface of the upper layer. After 2 to 3 weeks at 37°C and 5% CO_2_, foci were stained with 0.005% Crystal Violet, viewed and counted. A photograph of each Petri dish was taken with a Nikon Coolpix 995 and the relative number of colonies/dish was analysed using Image J (Image processing and analysis in Java: <http://rsb.info.nih.gov/ij/>). Cells of each clone were plated in quadruplet per experiment.

*In vitro wound healing and time lapse microscopy*

The clones were plated in duplicate in 24-well tissue culture plates that had flat bottoms and low-evaporation lids (Becton Dickinson; ref. 353047). Once confluency was reached, wounds were created by scraping the monolayers with 200 µl disposable plastic pipette tips. In some experiments, 5 µM or 10μM aphidicolin (Sigma) was added to the cells before creating the wound. Any cellular debris was removed by washing with PBS (Phosphate-Buffered Saline). The plates were placed in a chamber fixed to the robotized platform of an inverted microscope (Leica DMRIB) and maintained at 37°C with 5% CO_2_. Images (magnification ×40; Hoffman contrast) were collected every 20 min for 48 h with a Cool Snap FX camera using Metamorph software (Universal Imaging). The distance between the wound edges was measured using Adobe Photoshop CS2. To study the effects of ANO1 inhibition, 60 to 80% confluent cells were transfected with 25 nM siRNA (HEp-2 clones) or 50 nM siRNA (SCC-25 cells). The wounds were made when confluence was reached (24 h to 48 h after transfection). To study the effects of pharmacological inhibitors, the cells were seeded (1.2 x 10^5^) into 24-well plates, grown to confluency, wounded and washed with PBS. The wounded monolayers were then incubated in the presence of the compounds [NA, (niflumic acid, Sigma-Aldrich); DIDS (Disodium 4,4′-diisothiocyanatostilbene-2,2′-disulfonate, Sigma-Aldrich); DCPIB (4-[(2-Butyl-6,7-dichloro-2-cyclopentyl-2,3- dihydro-1-oxo-1H-inden-5-yl)oxy] butanoic acid, Tocris Biosciences); CFTRinh172 (4-[[4-Oxo-2-thioxo-3-[3-trifluoromethyl)phenyl]-5-thiazolidinylidene]methyl]benzoic acid, Tocris Bioscience); and Flx (fluoxetine, N-Methyl-3-[(4-trifluoromethyl)phenoxy]-3-phenylpropyla mine hydrochloride, Tocris Bioscience), the solvent (DMSO 0.1%) or neither, for 0, 8, 24, 36, and 48 h (as indicated) and photographed.

*Boyden chamber migration and invasion assays*

Cell migration assay was performed according to the manufacturer’s instructions (Collagen Quantitative Cell Migration assay; Chemicon International, Inc.). 80% confluent cells were starved in the appropriate serum free medium for 24 h, detached with 5 mM EDTA in phosphate-buffered saline (PBS) and then seeded (2.5×10^5^) in Boyden chambers coated with Bovine Serum Albumin (BSA) or collagen I (Col I). After 2 h 30 min at 37°C and 5% CO_2_, the cells that had passed through the matrix barrier were stained, and the eluent optical density (OD) was measured at 550 nm. Cell invasion assays (Chemicon International, Inc.) were similar, except that the cells were seeded (2.5×10^5^) in Boyden chambers coated with ECM matrix (Matrigel). After incubation for 5 h 30 min, cells that passed through the membrane were quantified. The cells of each clone were plated in duplicate per experiment.

*Cell adhesion assay*

Cells were trypsinized and plated at 10^6^ cells / well in 6-well plates. After incubation for 10 min at 37°C, the non adhering cells were collected with 2 PBS washes. Adherent and non adherent cells were counted using a Neubauer hemocytometer. The cells of each clone were plated in duplicate per experiment.

*Cell spreading assay*

Cell spreading assays were performed as described by (Rodrigu*es et* al, 2005). The cells of each clone were plated in duplicate at 2×10^5^ cells/well in 24-well plates with flat bottoms and low-evaporation lids. Images were collected every 5 min for 3 h at 20× magnification. The ratio of spreading cells relatively to the total number of adherent cells was calculated at different time points.

*Cell detachment assay*

Cell detachment assays were performed as described by (Tchou-Wo*ng et* al, 2006). The cells of each clone were seeded in duplicate in trypsin free medium, at a density of 2.5×10^5^ cells/well in 6 well-plates. After 24 h, the cells were washed once in diluted trypsin and incubated in fresh trypsin for 5 min at room temperature (RT) and 5 min at 37°C. All detached cells were collected with one wash with PBS. The detached cells and the remaining adherent cells were counted (Neubauer hemocytometer).

**Supplementary Tables**

**Supplementary Table 1**. Cox proportional hazard univariate analysis of the SNP logRratios against future metastasis (M) status, for probes located on chromosome 11 from 68Mb to 71Mb (corresponding to the peak of frequency of gain in 11q13), using the same HPV negative patient DNA samples as were used for CGH array analysis in our previous study (Rickm*an et* al, 2008). The columns correspond to the snp id; the p-value for the logrank test; the relative risk of metastasis; the chromosome; the chromosomal location in basepair; the closest gene symbol ; the closest entrez gene id ; the genome build. The table is ordered according to increasing logrank test p-values. The ANO1 (=TMEM16A) probe with the lowest p-value is highlighted in yellow.

**Supplementary Table 2**. Cox proportional hazard univariate analysis of the Affymetrix log2signal intensities, for the probe sets located on chromosome 11 from 68Mb to 71Mb and being assigned to a HUGO gene symbol, using the data for the HPV negative patients described in our previous study (Rickm*an et* al, 2008). The columns correspond to the probe set identifier, the logrank test p-value, the relative risk of metastasis, the gene symbol, the chromosome, the cytoband, the chromosomal location in basepair (start and end), the strand. The table is ordered according to increasing logrank test p-values. The ANO1 probe with the lowest p-value is highlighted in yellow.

**Supplementary Table 3**. Metastasis status of the sample's and available profiles for each platform (SNP, CGHa, expression). Column 1: Sample ID. Column 2: Metastasis status (0=No, 1=Yes). Column 3: available profile on 370K Illumina SNP (0=No,1=Yes). Column 4: available profile on 4.7K CGH BAC array (0=No,1=Yes). Column 5 : available profile on Affymetrix HGU133plus2.0 (0=No,1=Yes)

**Supplementary References**

Carles A, Millon R, Cromer A, Ganguli G, Lemaire F, Young J, Wasylyk C, Muller D, Schultz I, Rabouel Y, Dembele D, Zhao C, Marchal P, Ducray C, Bracco L, Abecassis J, Poch O, Wasylyk B (2006) Head and neck squamous cell carcinoma transcriptome analysis by comprehensive validated differential display. *Oncogene* **25:** 1821-31

Gentleman RC, Carey VJ, Bates DM, Bolstad B, Dettling M, Dudoit S, Ellis B, Gautier L, Ge Y, Gentry J, Hornik K, Hothorn T, Huber W, Iacus S, Irizarry R, Leisch F, Li C, Maechler M, Rossini AJ, Sawitzki G, Smith C, Smyth G, Tierney L, Yang JY, Zhang J (2004) Bioconductor: open software development for computational biology and bioinformatics. *Genome Biol* **5:** R80

Irizarry RA, Hobbs B, Collin F, Beazer-Barclay YD, Antonellis KJ, Scherf U, Speed TP (2003) Exploration, normalization, and summaries of high density oligonucleotide array probe level data. *Biostatistics* **4:** 249-64

Picard F, Robin S, Lavielle M, Vaisse C, Daudin JJ (2005) A statistical approach for array CGH data analysis. *BMC Bioinformatics* **6:** 27

Rickman DS, Millon R, De Reynies A, Thomas E, Wasylyk C, Muller D, Abecassis J, Wasylyk B (2008) Prediction of future metastasis and molecular characterization of head and neck squamous-cell carcinoma based on transcriptome and genome analysis by microarrays. *Oncogene* **27:** 6607-22

Rodrigues SP, Fathers KE, Chan G, Zuo D, Halwani F, Meterissian S, Park M (2005) CrkI and CrkII function as key signaling integrators for migration and invasion of cancer cells. *Mol Cancer Res* **3:** 183-94

Rouveirol C, Stransky N, Hupe P, Rosa PL, Viara E, Barillot E, Radvanyi F (2006) Computation of recurrent minimal genomic alterations from array-CGH data. *Bioinformatics* **22:** 849-56

Staaf J, Lindgren D, Vallon-Christersson J, Isaksson A, Goransson H, Juliusson G, Rosenquist R, Hoglund M, Borg A, Ringner M (2008a) Segmentation-based detection of allelic imbalance and loss-of-heterozygosity in cancer cells using whole genome SNP arrays. *Genome Biol* **9:** R136

Staaf J, Vallon-Christersson J, Lindgren D, Juliusson G, Rosenquist R, Hoglund M, Borg A, Ringner M (2008b) Normalization of Illumina Infinium whole-genome SNP data improves copy number estimates and allelic intensity ratios. *BMC Bioinformatics* **9:** 409

Tchou-Wong KM, Fok SY, Rubin JS, Pixley F, Condeelis J, Braet F, Rom W, Soon LL (2006) Rapid chemokinetic movement and the invasive potential of lung cancer cells; a functional molecular study. *BMC Cancer* **6:** 151

Yang YH, Dudoit S, Luu P, Lin DM, Peng V, Ngai J, Speed TP (2002) Normalization for cDNA microarray data: a robust composite method addressing single and multiple slide systematic variation. *Nucleic Acids Res* **30:** e15
